# Sole microbiome progression in a hatchery life cycle, from egg to juvenile

**DOI:** 10.3389/fmicb.2023.1188876

**Published:** 2023-06-26

**Authors:** Diana Bastos Almeida, Miguel Semedo, Catarina Magalhães, Isidro Blanquet, Ana Paula Mucha

**Affiliations:** ^1^ICBAS – Instituto de Ciências Biomédicas Abel Salazar, University of Porto, Porto, Portugal; ^2^CIIMAR - Interdisciplinary Centre of Marine and Environmental Research, University of Porto, Matosinhos, Portugal; ^3^SEA EIGHT - Safiestela S.A., Estela, Portugal; ^4^FCUP – Faculty of Sciences, University of Porto, Porto, Portugal

**Keywords:** host microbiome, core microbial community, senegalese sole, recirculating aquaculture system, amplicon sequencing

## Abstract

Recirculating aquaculture systems (RAS) pose unique challenges in microbial community management since they rely on a stable community with key target groups, both in the RAS environment and in the host (in this case, *Solea senegalensis*). Our goal was to determine how much of the sole microbiome is inherited from the egg stage, and how much is acquired during the remainder of the sole life cycle in an aquaculture production batch, especially regarding potentially probiotic and pathogenic groups. Our work comprises sole tissue samples from 2 days before hatching and up to 146 days after hatching (−2 to 146 DAH), encompassing the egg, larval, weaning, and pre-ongrowing stages. Total DNA was isolated from the different sole tissues, as well as from live feed introduced in the first stages, and 16S rRNA gene was sequenced (V6-V8 region) using the Illumina MiSeq platform. The output was analysed with the DADA2 pipeline, and taxonomic attribution with SILVAngs version 138.1. Using the Bray–Curtis dissimilarity index, both age and life cycle stage appeared to be drivers of bacterial community dissimilarity. To try to distinguish the inherited (present since the egg stage) from the acquired community (detected at later stages), different tissues were analysed at 49, 119 and 146 DAH (gill, intestine, fin and mucus). Only a few genera were inherited, but those that were inherited accompany the sole microbiome throughout the life cycle. Two genera of potentially probiotic bacteria (*Bacillus* and *Enterococcus*) were already present in the eggs, while others were acquired later, in particularly, forty days after live feed was introduced. The potentially pathogenic genera *Tenacibaculum* and *Vibrio* were inherited from the eggs, while *Photobacterium* and *Mycobacterium* seemed to be acquired at 49 and 119 DAH, respectively. Significant co-occurrence was found between *Tenacibaculum* and both *Photobacterium* and *Vibrio*. On the other hand, significantly negative correlations were detected between *Vibrio* and *Streptococcus*, *Bacillus*, *Limosilactobacillus* and *Gardnerella*. Our work reinforces the importance of life cycle studies, which can contribute to improve production husbandry strategies. However, we still need more information on this topic as repetition of patterns in different settings is essential to confirm our findings.

## 1. Introduction

Recirculating aquaculture systems (RAS) have been developed to reduce water usage through waste management, and so, making intensive fish production compatible with environmental sustainability (Piedrahita, 2003). However, these types of systems pose unique challenges in microbial community management, being extremely demanding to maintain a stable and healthy microbial community within the RAS environment (Schreier et al., 2010; Martins et al., 2013).

Microbiomes usually form specific communities in different physical and biological environments, with a dynamic and interactive nature crucial for the functioning and health of their hosts (Berg et al., 2020). Due to their dynamic nature, bacterial colonization in its host can be heavily influenced by diet and environmental conditions (Bledsoe et al., 2016; Wilkes et al., 2019). In fish, this translates, for example, in the role live feed plays in early development stages (Califano et al., 2017) as vectors for potential pathogenic bacteria of the genus *Vibrio* (Montanari et al., 1999; Olafsen, 2001). The gut microbiome has already been extensively studied due to its role in reinforcing the digestive and immune system of the fish (Talwar et al., 2018). The composition of the fish diet affects gut microbiome composition, thus different diets applied to the different stages of fish development are expected to influence gut microbial communities during its life cycle (Stephens et al., 2016). Because the live feed administered in the early stages of development is known for being relatively poor in nutrients, the richness of the fish diet is higher in later stages (with commercial feed), which is conflicting with the importance of early bacterial colonization (Yukgehnaish et al., 2020). Another factor contributing to an improvement in bacterial colonization is the transition to RAS systems, as the establishment of the fish microbiome can be affected by the change in environmental conditions, with fish developing different profiles after this period (Steiner et al., 2021).

There is a multiplicity of ecological processes in microbiomes that affect community assembly (Goldford et al., 2018), such as selective pressures and nutrient availability, which causes cross-feeding networks with microbes communicating and trading metabolites and services, especially relevant in anaerobic environments (Marx, 2009). On the other hand, competitive interactions may also play an important role in shaping host microbial communities (Coyte et al., 2015). In aquaculture, and RAS in particular, life cycle studies are still rare, although they are required to detect temporal changes of the microbiome along farming cycles to identify the core taxa for future modulation (Infante-Villamil et al., 2021).

As mentioned above, microbiome studies are important to better understand how pathogen outbreaks occur and identify dysbiosis events. The community in RAS, particularly in the biofilter (a sector for optimal but undifferentiated bacterial growth used for ammonia removal from the system), influences the farmed fish that is in constant contact with the water, with its own prokaryotic community (Laurent et al., 2000) that also provides continuity between different physical and biological environments (host and biofilter, for example). Therefore, in this complex and interactive environment, there is a risk that disruptions may cause pathogenic outbreaks by opportunist bacteria (Blancheton et al., 2013). Groups commonly associated with disease outbreaks in sole are the *Tenacibaculum* genus (Gourzioti et al., 2018), *Vibrio* (Austin, 2010) and *Photobacterium* (Toranzo et al., 2005). The first two have also been linked in a pathogenic dysbiosis event (Wynne et al., 2020). The family Mycobacteriaceae also includes a large number of pathogenic bacteria for a number of different fish species (Delghandi et al., 2020).

The prokaryotic community can also result in improved nutrition and effective disease control by inhibiting potential fish pathogens (Irianto and Austin, 2002). In aquaculture, several microbial species, mainly present in the fish gut and water, have already been identified as potentially probiotic with several health benefits such as improved fish productivity, resistance to diseases and increased immune functions (el-Saadony et al., 2021). Microbiome studies can then help to guide the best practices to promote the persistence of these agents (Borges et al., 2021). Some of the bacterial orders already identified as having potentially probiotic interest are Lactobacillales (Alonso et al., 2019) and Bifidobacteriales (Quigley, 2017). Additionally, the genera *Bacillus* (Kuebutornye et al., 2020), *Roseobacter*, *Phaeobacter*, *Paenibacillus*, *Pseudoalteromonas*, *Alteromonas*, *Pseudomonas*, *Aeromonas*, *Arthrobacter*, *Clostridium* (Ringø, 2020), *Saccharomyces* (Gaggìa et al., 2010), *Streptomyces* (Teng Hern et al., 2019), and *Shewanella* (García de la Banda et al., 2010)have also been linked to this activity.

Our goal in this paper is to start filling the gap on the microbiota analysis during fish life cycle in aquaculture. That is, to characterize the bacterial community along a farming cycle, accompanying a batch from egg to the pre-ongrowing stage. In this study we were able to evaluate the temporal microbiota progression across sole life cycle, providing a reference microbiota map for this species at different stages of development. In addition, we were able to determine how much of the sole microbiome is inherited from the egg stage, and how much is acquired in the different production stages. This work improved the background knowledge needed to develop future microbiome modulation in sole production. Additionally, the results presented here can have a direct impact in the production husbandry strategies.

## 2. Materials and methods

### 2.1. Sample collection

This study was performed in partnership with an aquaculture production unit, who provided the samples, a sole hatchery (Safiestela S.A.), located in Estela, Portugal. The pre-ongrowing and weaning tanks operate in a recirculating aquaculture system (RAS), while egg and larval stages are kept in a flow-through water system. The water circulation of the pre-ongrowing (PO) and weaning (WE) systems is displayed in Supplementary Figure S1 and it was previously described (Almeida et al., 2021). Briefly, after circulating through the tanks, wastewater is mechanically filtered with a rotary drum filter (mainly for particulate organic matter removal), followed by biological filtration with a moving bed biofilter reactor type (volume of 150 m^3^ in the PO and 25 m^3^ in the WE system). After the degasification column, where water trickles down, the water passes through the skimmer before returning to the tanks. The total water volume is 370 m^3^ in the PO system and 60 m^3^ in the WE system. In both systems, the water recirculation rate is approximately 400% per hour, the feeding regime is approximately 2% biomass/day, and the fish density varies between 2.5 to 5 kg/m^2^.

**Figure 1:**
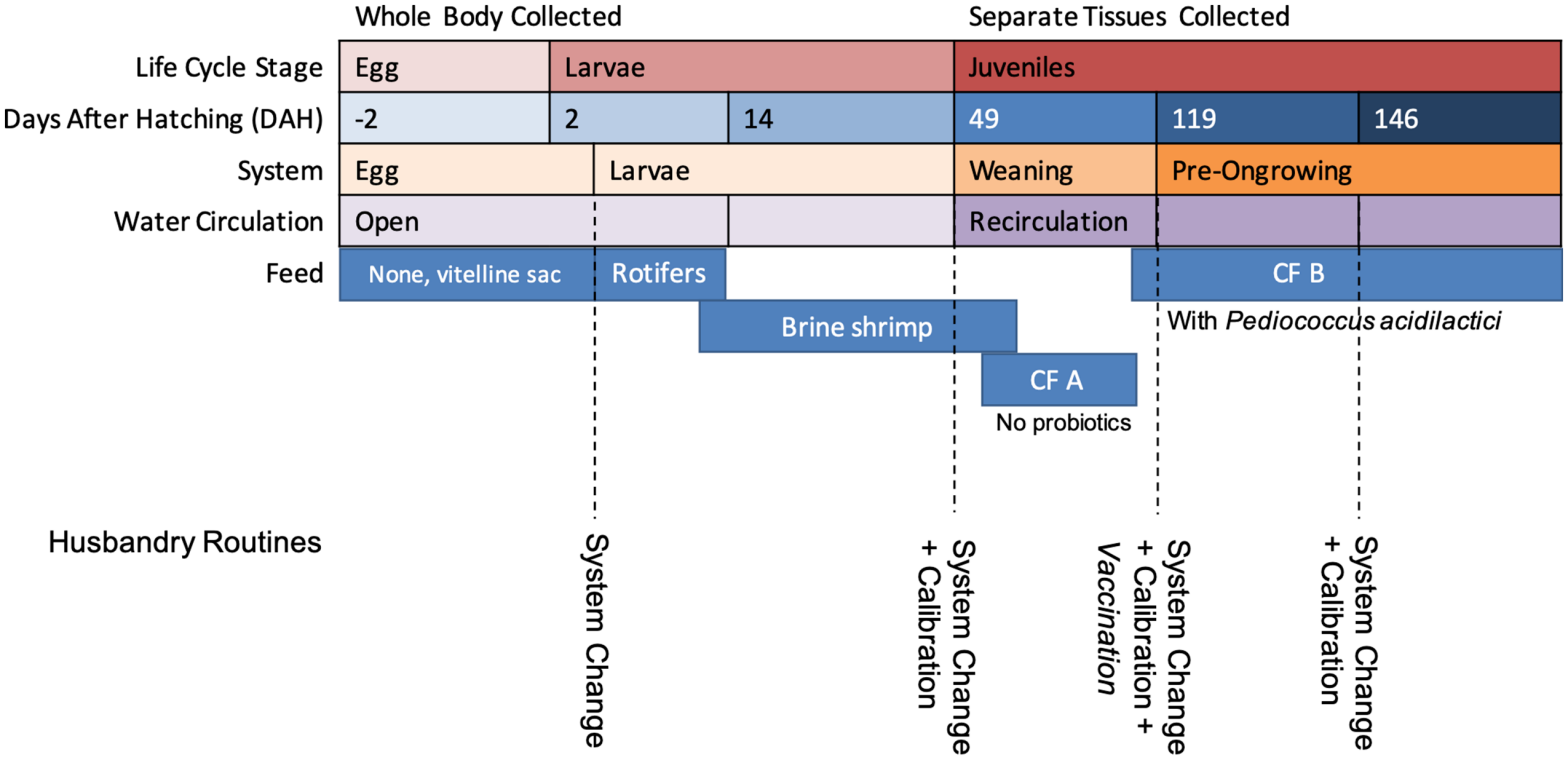
A resume of the age (days after hatching) at which fish samples were collected, the system they were collected from, and the life cycle stage associated and feed.

A description of the age, system, life cycle stage, and feed of the collected samples is presented in Figure 1. Fish larvae were fed rotifers from 2 to 5 days after hatching (DAH) and brineshrimp from 7 to approximately 75 DAH (slightly after entering the WE system). Commercial feed (*CF*) A, for flatfish larvae with no potentially probiotic added, was introduced at 65 DAH and replaced by *CF* B, for nursery with supplemented potentially probiotic *Pediococcus acidilactici*, at 100 DAH. The exact amount of *P. acidilactici* was not disclaimed in the commercial diet formulation, but the BACTOCELL CNCM I-4622 strain was used. For this study, the same production batch was accompanied throughout the development stages and tissue samples were collected in duplicate. Eggs were collected at −2 DAH, larvae at 2 and 14 DAH. For juveniles, the separate tissues were collected for microbiome characterization (caudal fin, gills, mucus and intestine) at the weaning system (49 DAH) and at the beginning and end of the pre-ongrowing (119 and 146 DAH, respectively). For each sample type, on each day, duplicate samples were collected, one fish per sample in the case of the juveniles, and approximately 2 mL of dry volume in the case of egg and larvae samples. Live feed samples were also collected in duplicate. Information about temperature, salinity, and pH at the sampling time can be found in Supplementary Table S1.

### 2.2. DNA extraction and sequencing

Total DNA was isolated from the different matrices (eggs, larvae, caudal fin, gills, mucus and intestine, live feed), in duplicate, with DNeasy Power Soil kit (QIAGEN, Merck KGaA, Darmstadt, Germany). Samples were prepared for Illumina Sequencing by 16S rRNA gene amplification of the bacterial community. The DNA was amplified for the hypervariable V6-V8 region with specific primers and further reamplified in a limited-cycle PCR reaction to add sequencing adapters and dual indexes. First PCR reactions were performed for each sample using KAPA HiFi HotStart PCR Kit according to manufacturer suggestions, 0.3 μM of each PCR primer: forward B969F 5′- ACGCGHNRAACCTTACC -3′ and reverse BA1406R 5′- ACGGGCRGTGWGTRCAA −3′ (Michl et al., 2019) and 50 ng of template DNA in a total volume of 25 μL. The PCR conditions involved a 3 min denaturation at 95°C, followed by 35 cycles of 98°C for 20 s, 60°C for 30 s and 72°C for 30 s and a final extension at 72°C for 5 min. Second PCR reactions added indexes and sequencing adapters to both ends of the amplified target region according to manufacturer’s recommendations. Negative PCR controls were included for all amplification procedures. PCR products were then one-step purified and normalized using SequalPrep 96-well plate kit (ThermoFisher Scientific, Waltham, United States) (Comeau et al., 2017), pooled and pair-end sequenced in the Illumina MiSeq^®^ sequencer with the V3 chemistry, according to manufacturer’s instructions (Illumina, San Diego, CA, United States) at Genoinseq (Cantanhede, Portugal).

### 2.3. Sequence processing and analysis

To obtain a amplicon sequence variant (ASV) table, the DADA2 pipeline (Callahan et al., 2016) was implemented on our dataset. This was done using the R environment (version 4.1.2. *Copyright 2019, the R Foundation for Statistical Computing*) with the package *dada2* (v1.16.0). Primer removal was performed within the pipeline of DADA2 using the filterAndTrim function. Sequence filtering, trimming, error rates learning, dereplication, chimera removal and amplicon sequence variant (ASV) inference were performed with default settings. For taxonomic attribution, the SILVAngs version 138.1 database was used (Quast et al., 2013). Taxa classified at the kingdom level as Eukaryota, at the order level as Chloroplast and at the family level as Mitochondria were removed.

For the general bacterial community analysis, the package *phyloseq* (v1.38.0) and *ggplot2* (v3.3.5) were used for data handling and visualization. Alpha diversity was calculated using the Observed ASVs metric and the Shannon index with *vegan* (v2.5–7). Beta-diversity was calculated with Bray–Curtis dissimilarity index and plotted with non-metric multidimensional scaling (NMDS), this was also performed for the target groups subsets (potentially pathogenic and potentially probiotic). Dissimilarity results were tested by permutational multivariate ANOVA (PERMANOVA) using the Adonis function (vegan) for beta group significance (*p*-values lower than 0.05) the parameters age (DAH), sample type (egg, larvae, fin, gills, mucus and intestine), life cycle stage (egg, larvae, juveniles) and system (egg, larvae, weaning, pre-ongrowing) were tested.

To be part of the core microbiome, we consider the bacterial genera that are present in at least 75% of all samples of the sole life cycle (prevalence), with an abundance higher than 0% (detection threshold), using the *microbiome* R package (v. 1.16.0). Additionally, venn diagrams were performed to analyze the membership of shared taxa across the sole life cycle with tissue samples were separated by life cycle stages. Venn diagrams were obtained using the *venn* R package (v. 1.10) to display the number of shared and exclusive taxa between whole body samples (egg and larvae) and each sole tissue (fin, gill, intestine, mucus) at different ages (49, 119, and 146 days).

To explore our target groups, potentially probiotic and potentially pathogenic bacterial organisms, these groups were identified at different taxonomic levels to mitigate the effects of unclassified sequences and (in the case of probiotics) to potentially find new promising genera for further studies. For the potentially probiotic group, we selected all genera from the order Lactobacillales (Alonso et al., 2019) and Bifidobacteriales (Quigley, 2017), and also the genera *Bacillus, Roseobacter*, *Phaeobacter*, *Paenibacillus*, *Pseudoalteromonas*, *Alteromonas*, *Pseudomonas*, *Aeromonas*, *Arthrobacter*, *Clostridium* (Ringø, 2020), *Saccharomyces* (Gaggìa et al., 2010), *Streptomyces* (Teng Hern et al., 2019), and *Shewanella* (García de la Banda et al., 2010), due to their previously identified role in probiotic potential and activity. The genera *Tenacibaculum* (Gourzioti et al., 2018), *Vibrio* (Austin, 2010), *Photobacterium* (Toranzo et al., 2005) and *Mycoplasma* (Delghandi et al., 2020) were selected as potentially pathogenic as it was demonstrated in previous studies. Notwithstanding the potential of our selected taxonomic groups to contain probiotic or pathogenic species, we must acknowledge that some of these genera also contain non-probiotic or non-pathogenic organisms. Thus, one cannot infer direct fish health effects (probiotic or pathogenic) from the detection of these groups in our study. When deciding where to categorize these genera groups, we considered as pathogenic only those previously associated with disease outbreaks in *Solea senegalensis*. For the potential probiotic list, we gathered those with probiotic activity described in the literature that had not yet been described as pathogenic for *Solea senegalensis*. A correlation matrix between the relative abundance of our target groups was also built with significant correlations (Spearman pairwise, value of *p* <0.05) using the R packages *Hmisc* (v4.1.1) and *corrplot* (v0.84).

### 2.4. Ethics declaration and data availability

The animals used in this work were not subjected to any experimental protocol and were a part of the routine procedures of a commercial hatchery facility. All animals were handled by the fish farm employees, the euthanasia method used was an anaesthetic overdose of the commercial anaesthetic Aquacen benzocaine 200 mg/mL (CENAVISA, S.L., Spain), according to manufacturer instructions, and following SEA EIGHT’s Veterinary Plan. According to the Portuguese legislation DL N° 113/2013, this work is exempted from the need for ethical approval. All methods are reported in accordance with ARRIVE guidelines.

The datasets generated and/or analysed during the current study are available in the European Nucleotide Archive (ENA) repository, accession number PRJEB55703.

## 3. Results

The 16S rRNA gene sequencing dataset used had a minimum and maximum read counts per sample (after trimming) of 7,776 and 84,097, respectively. The mean read counts for all samples was 32,709, the complete list of read counts per sample is presented in Supplementary Table S2.

### 3.1. General bacterial community

**Figure 2:**
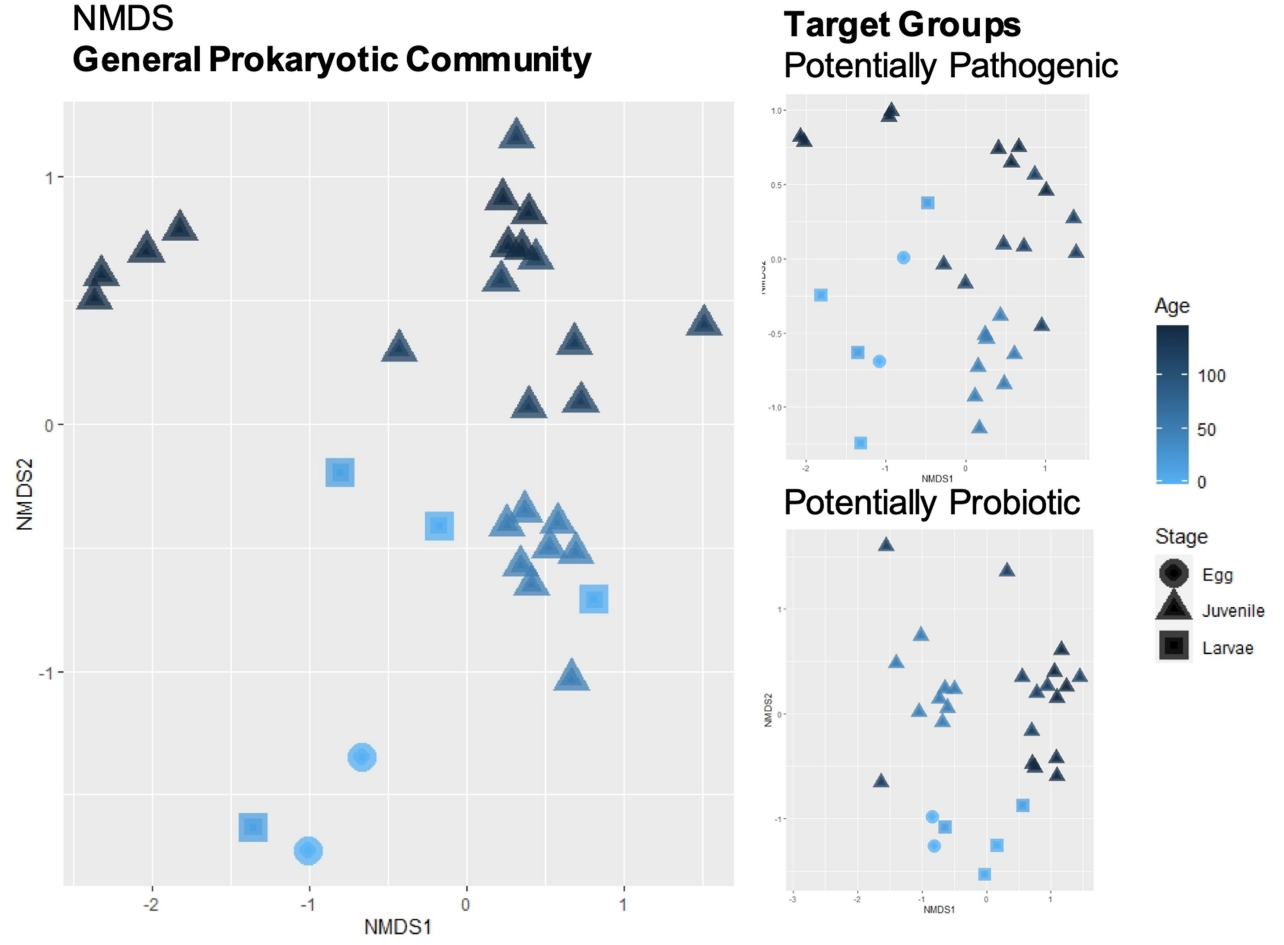
Beta-diversity calculated with Bray–Curtis dissimilarity index and plotted with non-metric multidimensional scaling (NMDS) was performed for the general prokaryotic community and for the subsets of the target groups (potentially pathogenic and potentially probiotic). Sample shapes represent the life cycle stage of the fish and gradient color represents their respective age.

The most abundant phyla were Proteobacteria (42–91%), Bacteroidetes (or Bacteroidota, 2–40%) and Firmicutes (0–39%). The complete distribution at this taxonomic level can be found in Supplementary Figure S2 and at the genus level (abundance >1%) in Supplementary Figure S3 (Supplementary Table S3 for an ASV breakdown of abundances). Overall, alpha diversity indexes did not appear to be influenced by the different phases of the sole life cycle or type of tissue at the juvenile stage (Supplementary Figure S4 and Supplementary Table S2). The NMDS distribution of the Bray–Curtis dissimilarity index had a stress value of 0.166 and is plotted in Figure 2. It shows an apparent grouping by age and life cycle stage. All the parameters tested, age (DAH), sample type (egg, larvae, fin, gills, mucus and intestine), life cycle stage (egg, larvae, juveniles) and system (egg, larvae, weaning, pre-ongrowing), had significant *p*-values in the Adonis test, with the system having the highest percent variability explained (Supplementary Table S4). However, only the life cycle stage had a non-significant homogeneity of dispersion test. The % variability explained and the dispersion test indicate that both “system” and life-cycle” were the most important factors in shaping community dissimilarity.

**Figure 3:**
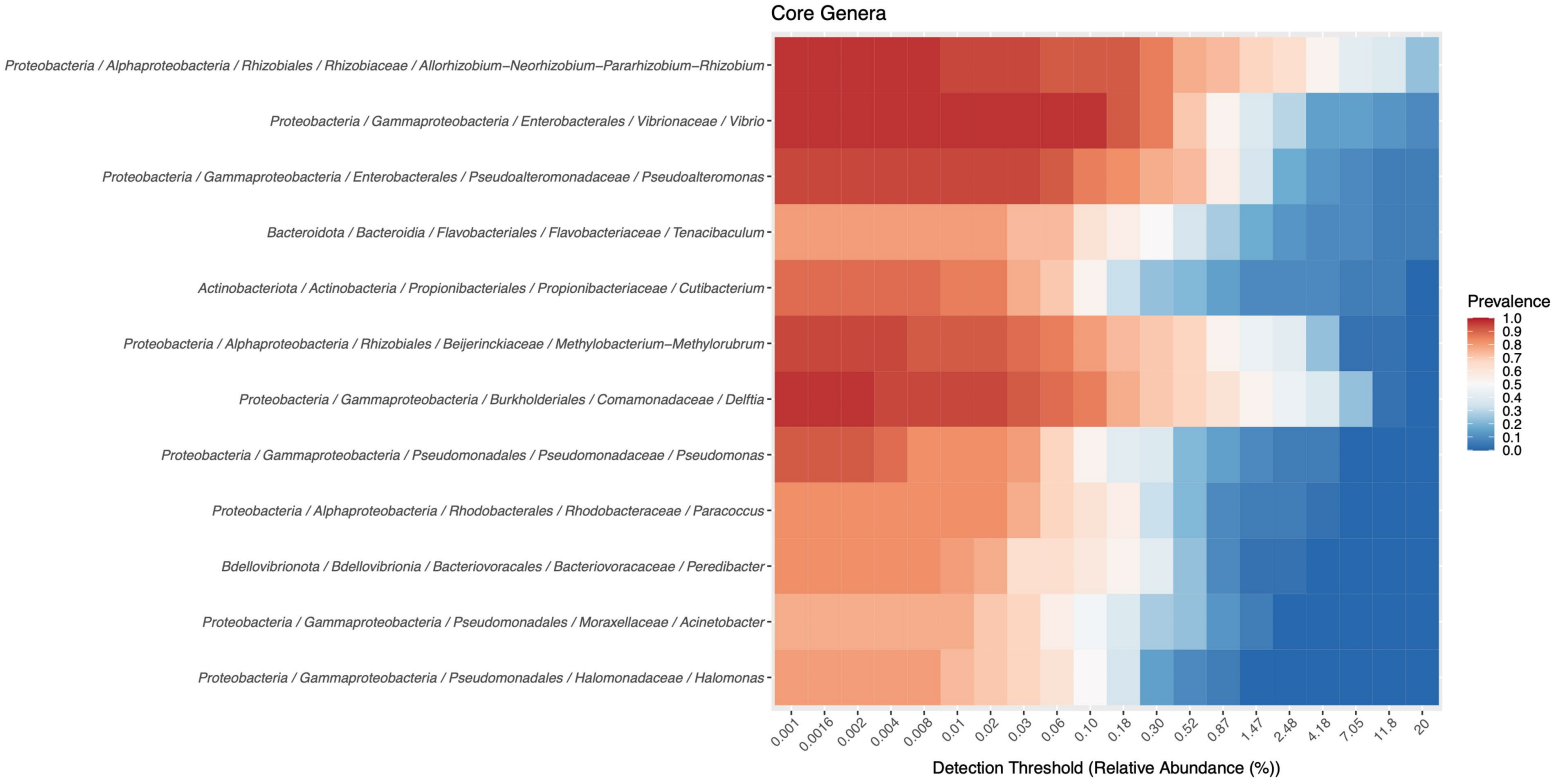
Members of the core microbiota were determined with a detection threshold of 0 and a prevalence threshold of 0.75.

The core microbiome, at the genus level, can be consulted in Figure 3. Twelve genera are part of this core microbiome, *Allorhizobium-Neorhizobium-Pararhizobium-Rhizobium*, *Vibrio*, *Pseudoalteromonas, Tenacibaculum, Cutibacterium*, *Methylobacterium*-*Methylorubrum*, *Delftia*, *Pseudomonas*, *Paracoccus*, *Peredibacter*, *Halomonas,* and *Marinobacter*.

**Figure 4:**
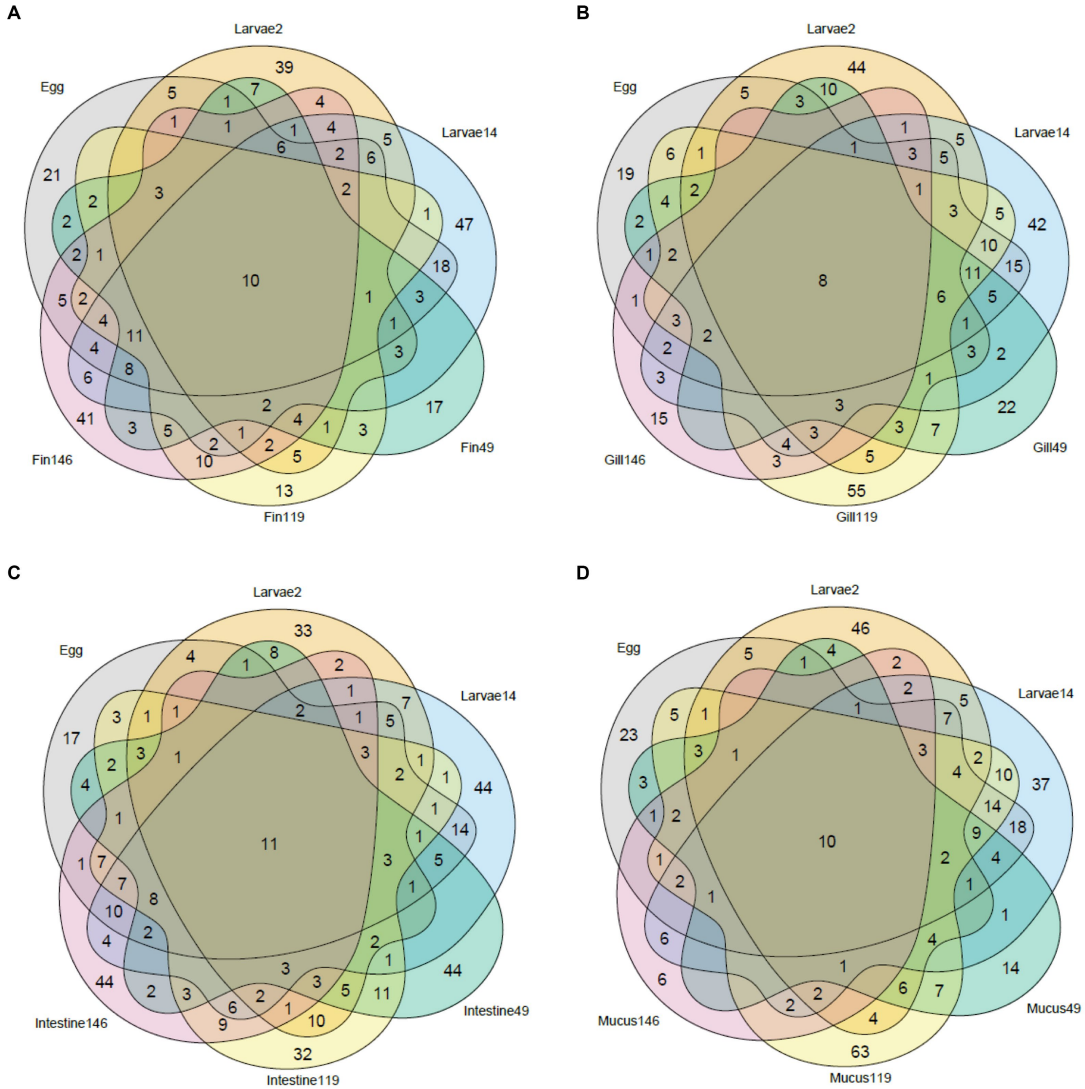
Venn diagram of the shared taxa between whole body samples (Egg and Larvae) and different types of tissue collected from later stages of the sole: fin **(A)**, gill **(B)**, intestine **(C)**, and mucus **(D)**.

Venn diagrams (Figure 4) were used to distinguish the inherited from the acquired community along the sole life cycle, by analyzing the shared genera across sample types. In the caudal fin bacterial community, there are ten genera (that represent 2.9% of the genera in this analysis) that are present across the entire life cycle (*Allorhizobium-Neorhizobium-Pararhizobium-Rhizobium, Cutibacterium, Delftia, Halomonas, Marinobacter, Methylobacterium-Methylorubrum, Pseudoalteromonas, Sulfitobacter,* Unclassified *Cryomorphaceae, Vibrio*). In the gills, there are eight genera (2.2%) present across all samples (*Halomonas, Marinobacter, Phaeobacter, Pseudoalteromonas, Roseovarius, Tenacibaculum, Unclassified Cryomorphaceae, Vibrio*). A total of eleven genera (2.7%) were present across all intestinal samples (*Allorhizobium-Neorhizobium-Pararhizobium-Rhizobium, Colwellia, Cutibacterium, Delftia, Methylobacterium-Methylorubrum, Octadecabacter, Pseudoalteromonas, Roseovarius, Tenacibaculum, Vibrio, Yoonia-Loktanella*) and ten genera (2.9%) in the mucus (*Allorhizobium-Neorhizobium-Pararhizobium-Rhizobium, Delftia, Halomonas, Marinobacter, Methylobacterium-Methylorubrum, Pseudoalteromonas, Roseovarius, Tenacibaculum, Vibrio, Yoonia-Loktanella*). There is no apparent trend in the number of exclusive genera per tissue, with numbers varying between ages.

### 3.2. Target bacterial groups

**Figure 5:**
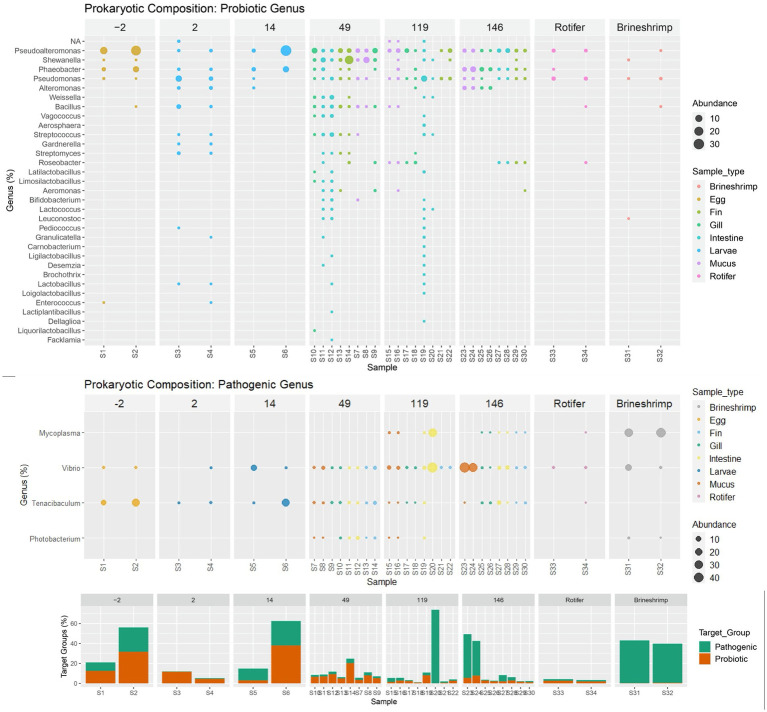
Relative genus distribution of the target groups (probiotic and potentially pathogenic) ordered by age and colored by sample type (Egg, Larvae, Gill, Intestine, Mucus, Fin, Rotifer or Brineshrimp) and with a bar plot summary of overall target group composition by sample. Samples with no detectable abundancy of each functional group have been removed.

Relative abundance of genus distribution of the target groups can be seen in Figure 5 and Supplementary Table S5. For the genera associated with potentially probiotic bacteria, it was observed that sequences from *Bacillus*, *Enterococcus, Phaeobacter, Pseudoalteromonas, Pseudomonas and Shewanella* were already present in the eggs (2 days before hatching). *Shewanella* disappears at 2 DAH and was only detected again in the WE system (at 49 DAH), after the two sources of live feed (rotifer and brineshrimp) were introduced. *Bacillus* and *Enterococcus* also drop below the detection limit (no sequences obtained) at 14 DAH, and only the first re-emerges in the WE system. The remaining three genera (*Pseudoalteromonas*, *Phaeobacter*, and *Pseudomonas*) were present throughout the life of the sole in the hatchery. Results showed that more potentially probiotic genera were introduced after hatching, at 2 DAH. At this stage of the life cycle the genera *Alteromonas*, *Streptococcus*, *Gardnerella*, *Streptomyces*, *Pediococcus*, *Granulicatella*, *Lactobacilus* emerged, but only the first was detected at 14 DAH (aside from *Phaeobacter, Pseudoalteromonas, Pseudomonas*). However, most of the other (except for *Gardnerella*) re-emerged in the WE system (at 49 DAH), around forty days after live feed was introduced. At this stage, 19 new genera appeared for the first time: *Weissella*, *Vagococcus*, *Aerosphaera*, *Roseobacter*, *Latilactobacillus*, *Limosilactobacillus*, *Aeromonas*, *Bifidobacterium*, *Lactococcus*, *Leuconostoc*, *Carnobacterium*, *Ligilactobacillus*, *Desemzia*, *Brochothrix*, *Loigolactobacillus*, *Lactiplantibacillus*, *Dellaglioa*, *Liquorilactobacillus* and *Facklamia*. At 146 DAH only 7 potential potentially probiotic genera were detected: *Pseudoalteromonas, Shewanella, Phaeobacter, Pseudomonas, Alteromonas, Roseobacter and Aeromonas*. Despite no major changes observed in total relative abundance of potentially probiotic genera, the number of detected genera increased from four at the end of the larval stage (14 DAH) to 22 and 21, in the intestine at day 49 and 119 respectively, and then back to 4 at 146 DAH.

In respect to the potentially pathogenic genera, *Tenacibaculum* and *Vibrio* accompany the sole microbiome through its development, from egg to 146 DAH. *Photobacterium* and *Mycoplasma* were detected, respectively, at 49 DAH and 119 DAH. *Photobacterium* was also detected in brineshrimp and *Mycoplasma* was detected in both brineshrimp and rotifer samples.

**Figure 6:**
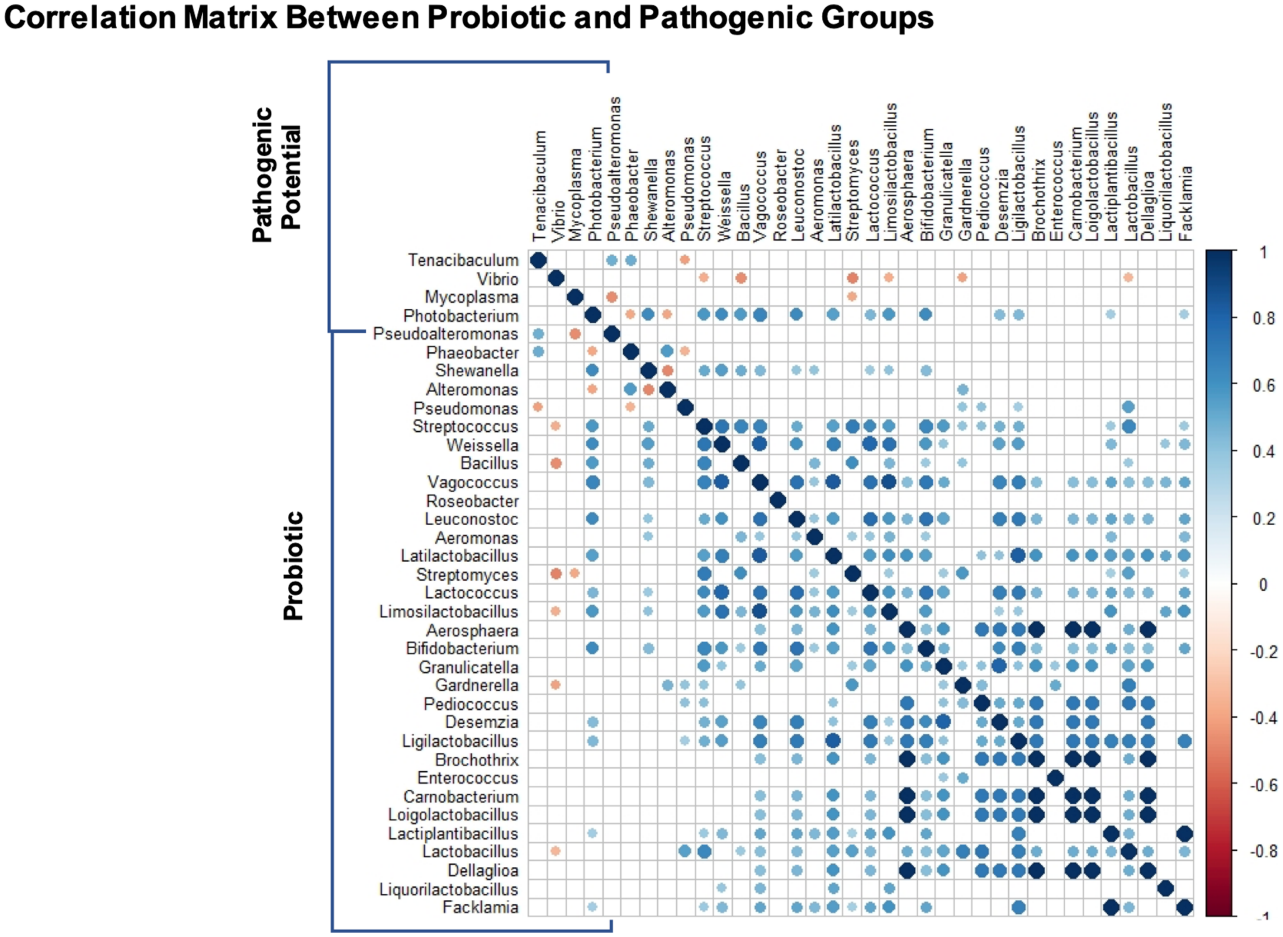
Genus-genus interactions between target groups: potentially probiotic and potentially pathogenic. The correlation matrix represents significant interactions (*p* < 0.05) using Spearman pairwise correlation coefficient.

The spearman correlation matrix between the relative abundances of potentially probiotic and pathogenic genera can be found in Figure 6. There are no significant correlations between the potentially pathogenic genera. Almost all correlations between potentially probiotic taxa are positive, despite two exceptions (*Alteromonas* with *Shewanella* and *Pseudomonas* with *Phaeobacter*). Regarding interactions across the two target groups, there are two positive correlations between *Tenacibaculum* and potentially probiotic taxa (*Pseudoalteromonas* and *Phaeobacter*) and one negative with *Pseudomonas*. There are only negative correlations between *Vibrio* and six potentially probiotic taxa (*Streptococcus*, *Bacillus*, *Streptomyces*, *Limosilactobacillus*, *Gardnerella* and *Lactobacillus*). Two potentially probiotic taxa have negative correlations with *Mycoplasma*, *Pseudoalteromonas* and *Streptomyces*. Finally, *Photobacterium* has two negative correlations with *Phaeobacter* and *Alteromonas* and 14 positive correlations with potentially probiotic taxa (*Shewanella*, *Streptococcus*, *Weissella*, *Bacillus*, *Vagococcus*, *Leuconostoc*, *Aeromonas*, *Lactococcus*, *Limosilactobacillus*, *Bifidobacterium*).

## 4. Discussion

Recirculating aquaculture systems have a unique challenge in managing a stable and functional microbial community (Schreier et al., 2010; Martins et al., 2013), with communities that are crucial for the health of the host (Berg et al., 2020) that can be heavily influenced by diet and environmental condition (Bledsoe et al., 2016; Wilkes et al., 2019). To fill the gap in life cycle studies, crucial to improve microbiome managing strategies, we characterized the bacterial community along a farming cycle, from egg to pre-ongrowing juveniles, evaluating the temporal microbiota evolution.

We found that alpha-diversity indexes did not change throughout development, although previous studies on a different species (Atlantic cod), refer to a loss of bacterial species diversity when artificial feeding is introduced (Ringø et al., 2006). Besides the fish species difference, the mentioned study was also technically very different, since the bacterial diversity was explored through isolation (Ringø et al., 2006), possibly missing the difficult to cultivate members of the community. The lack of substantial changes in bacterial alpha-diversity observed over the course of our study, is worth underscoring since it suggests a crucial importance of the early stages of fish development in establishing microbial community diversity.

The most abundant phyla in this dataset, Proteobacteria, Bacteroidetes and Firmicutes are commonly found to be the most abundant in aquaculture systems (Bledsoe et al., 2016; Wilkes et al., 2019). At the genus level, it appears that there are no dominating genera across the bacterial community and there is some variability in the relative abundance of genera detected between duplicates of the same sample. This variability in the bacterial community composition may be a consequence of the formation of heterogenous physical and biological micro-environments within the fish host, with specific bacterial communities as described by other studies (Zhang et al., 2019; Sylvain et al., 2020).

The term “core microbiome” has become widely used in microbial ecology to describe the set of microbial taxa that characterize a host or environment of interest (Neu et al., 2021). In this work we used a shared core microbiome analysis to infer possible conserved ecological roles and found that it was composed of twelve genera, four of them (*Allorhizobium-Neorhizobium-Pararhizobium-Rhizobium*, *Vibrio*, *Pseudoalteromonas* and *Tenacibaculum*) present in all tissues analysed and in all growth stages. As mentioned before, two of them are potentially pathogenic (*Vibrio* and *Tenacibaculum*). One thing to keep in mind is that both the live feed and the border sole tissues collected are in permanent contact with the water, and when studying these frontier environments its complex to disentangle the host from the environment community. Indeed, three of these genera have already been identified in the water, tank biofilm and biofilter carriers in this aquaculture unit, *Vibrio*, *Pseudoalteromonas* and *Tenacibaculum* (Almeida et al., 2021).

Using Venn diagrams, we found that the inherited community had very few genera represented (2.2–2.9%), all of them included *Tenacibaculum* and *Vibrio*. However, to our knowledge, this is the first time this type of characterization is performed in an aquaculture setting. Studies that accompany the evolution of the microbiome are rare, although there are some studies that accomplished a similar characterization, but only in wild populations. In migratory wild salmon, there is a microbiota community destabilization in migratory phases of the life cycle (Llewellyn et al., 2016). Another study found that, although deep-sea anglerfish microbiomes are dominated by the same genera from larvae to adult, their characteristic bacterial bioluminescent symbionts were not present in the early stages and were acquired from the environment (Freed et al., 2019).

Two target groups were selected (potentially pathogenic and probiotic) as having the most impact during the sole life cycle in an aquaculture production batch. We found a sharp increase in the number of potentially probiotic genera when sole moved to the WE system (49 DAH), around 40 days after live feed was introduced in the diet. Commercial feed did not appear to substantially increase the number of potentially probiotic genera at 119 or 146 DAH. Despite carrying *Pediococcus acidilactici* in its formulation, the commercial feed B did not consistently increase the abundance of *Pediococcus* in the sole intestines fed with this diet (119 and 146 DAH). When considering the increased number of potentially probiotic genera in the fish tissues after day 49, it is worth noticing that most of these genera were not present in the feed itself (live or commercial). An explanation could be that components in these feeds may act as prebiotics, that is, nutrients that are not digested by the fish that may fortify certain components of the intestinal microbiota by stimulating the growth and the activity of particular bacteria (Ringø et al., 2010). Indeed, prebiotic supplementation has shown potential as a strategy to overcome chronic stress-induced disease susceptibility in farmed *S. senegalensis* (Azeredo et al., 2019). Although reaching its highest number at 119 DAH, the number of potentially probiotic genera drops abruptly at 146 DAH with no change in the feed, raising the question if it was a consequence of husbandry or an unsuccessful establishment of the potential probiotic community. We should note, also, that most of the prokaryotic diversity is found in the rare biosphere (Pascoal et al., 2020), a genetic pool mostly undetected with the sequencing depth applied in this study, and some rare taxa can remain rare while others may grow abundant when the conditions change. This seed bank can include low abundant pathogenic communities and its monitorization could be useful in early identification, but can also support host functions specific to the aquaculture environment (Pascoal et al., 2022). This shift from undetectable to detectable groups may happen when the production alters the diet (specially between feeds), as the nutrients available change, diversity of certain genera increases momentarily and then declines with the stabilizing environmental conditions, explaining the drop of potentially probiotic genera at 146 DAH. Much like in the human gut microbiome, a diverse diet provides a competitive advantage to low abundant taxa, and the more diverse the microbiome, the more adaptable it will be to perturbations (Heiman and Greenway, 2016). Studies in chinook salmon also found that the gut microbiome is shaped by the environment, both by water and by formulated feed (Steiner et al., 2021). However, high inter-individual variation suggests that the host physiology itself may affect the community structure as much as environmental conditions (Fossmark et al., 2021; Hossain et al., 2021). In our study, it has also been observed that some genera associated with nitrifying activity (e.g., *Nitrosomonas* and *Nitrospira*) increased their relative abundances when fish were introduced to RAS, during the pre-ongrowing stage (Supplementary Table S5). Other studies have also found colonization of this group in fish tissue under similar conditions (van Kessel et al., 2016). Most probably, this is a consequence of nitrifying groups circulating from biofilters to the different compartments of the RAS unit, where they were found to occur (Almeida et al., 2021).

For the potentially pathogenic genera, *Tenacibaculum* and *Vibrio,* they appear to be acquired at the egg stage, accompanying the sole microbiome through its development, from egg to 146 DAH. The other two, *Photobacterium* and *Mycoplasma*, appear to be detectably colonizing later in the life cycle. In this study they have been identified in the rotifers and brineshrimp and thus the live feed could be a potential vector as has been previously demonstrated (Hurtado et al., 2020). This early diet driven microbiome development can have a significant impact in the future fish microbiome (Wilkes et al., 2019). Differentiating which pathogenic genera are inherited from those that the fish acquires throughout production is paramount. By identifying where in the production, the fish is exposed to these groups, husbandry improvements can be implemented to control them. However, if these pathogens are inherited from a wild broodstock, it may be difficult to safely remove them in a sustainable way. However, it is important to have in mind that the genera included in this study are potentially pathogenic, but are not composed solely by pathogenic species. In fact, the genus *Vibrio* is an important ecological marker, as it is widely abundant in riverine, estuarine, and marine aquatic environment (Hurtado et al., 2020) and one of the most diverse marine bacterial genera (Gomez-Gil et al., 2014). In the case of *Tenacibaculum*, out of 28 total species (Parte et al., 2020), only seven are generally associated with disease outbreaks: *T. maritimum, T. soleae, T. discolor, T. gallaicum, T. dicentrarchi, T. finnmarkense, T. ovolyticum* (Fernández-Álvarez and Santos, 2018). There are technologies available that may help to increase the definition for pathogenic species identifications. For example, long read sequencing with Pacific Biosciences (PacBio) or Oxford Nanopore Technology (ONT). These technologies had recent advancements that now provide higher accuracy and can provide up to 60 kb reads (Hu et al., 2021), almost enabling the sequencing of the complete 16S rRNA gene. Additionally, after the detection of groups of interest by an overall sequencing approach, like the one implemented in this study, targeted approaches to detect pathogenic markers of a subset of species could be implemented in a production setting to provide confirmation of pathogenicity. However, this was out of the scope of this work, which aimed at understanding the microbial progression along a fishery life-cycle, and not the occurrence of disease/stress.

In the correlation matrix, we found that six genera with potential probiotic activity were significantly negatively correlated with *Vibrio*, two of them, *Bacillus* and *Streptomyces* have already been described as potential inhibitors of *Vibrio* pathogen species (Vaseeharan and Ramasamy, 2003; Teng Hern et al., 2019). Only one genus had a negative correlation with *Tenacibaculum* (*Pseudomonas*), two with *Mycoplasma* (*Pseudoalteromonas*, *Streptomyces*) and two with *Photobacterium* (*Phaeobacter* and *Alteromonas*). For this analysis, we must recognize the potential biases in NGS community correlation studies that may result in misleading positive correlations, derived from the fact that more taxa are detected in deeply sequenced samples and therefore taxa co-vary with sequencing depth (Faust et al., 2015). Attesting to this, the correlation matrix shows a positive interaction between *Shewanella* and *Photobacterium*, however it had been amply reported that the first increases resistance to the later (García de la Banda et al., 2010, 2012; Vidal et al., 2016). These detected correlations can be useful to unveil possible interactions, but more studies are needed to confirm or discard mechanistic hypotheses. Also, it is relevant to note that *Photobacterium* has a total of 14 positive correlations with potentially probiotic taxa, which might also be a consequence of the positive bias. A similar observation occurred between *Phaeobacter* and *Tenacibaculum* (Edward et al., 2022). With the limits of these techniques, it is unreliable to distinguish between positive bias from non-specific potentially probiotic activity with positive correlations between pathogenic and potentially probiotic taxa in our data (*Phaeobacter* and *Vibrio*) or cases like *Bacillus* that shows a negative correlation with *Vibrio* but a positive one with *Photobacterium*. The genera *Streptococcus*, *Phaeobacter* and *Limosilactobacillus* also have a similar behavior. Four genera had exclusive positive correlations with the potential pathogenic bacteria (*Alteromonas*, *Pseudomonas*, *Gardnerella*, *Lactobacillus*), therefore these might be the most promising for future empirical studies.

## 5. Conclusion

This work aimed to describe the sole microbiome development throughout the production cycle in a RAS. Through a description of the inherited and acquired community in the different tissues analysed at different production and life stages, we hope to promote the emergence of life cycle studies in aquaculture and to underscore its applicability. We found that the bacterial community was significantly altered throughout the *Solea senegalensis* early development. Two potentially probiotic genera were inherited from the egg stage (*Bacillus* and *Enterococcus*), but the main increase in potentially probiotic abundance and diversity occurred around 40 days after live feed was introduced in the diet (at the weaning stage). Notwithstanding this increase, the establishment of this community in the following development stages was not successful. Regarding potentially pathogenic genera, two appear to be inherited (*Tenacibaculum* and *Vibrio*), and two are suggested to be acquired during production (*Photobacterium* and *Mycoplasma*). These results are relevant, because acquired potentially pathogenic groups may be prophylactically treated with improvement in husbandry conditions, but those that are inherited from the egg stage may be difficult to safely eradicate.

Our study has conducted a comprehensive description of the bacterial community in different life cycle stages of the *Solea senegalensis*, to our knowledge, the first of its kind. By analyzing the composition of this community, particularly with the definition of key target groups and the definition of the inherited and acquired community in the production cycle, we have highlighted the importance of whole life cycle studies to understand the vulnerability of the stages of fish production with a direct impact in husbandry strategies. The shifts in the composition of key components of *Solea senegalensis* gut microbiome during its life cycle, open important questions related to the functional significance of the observed taxonomic changes in terms of potentially probiotic activity and pathogenic incidences in the life cycle of this fish that must be explored in future investigations.

## Data availability statement

The datasets presented in this study can be found in online repositories. The names of the repository/repositories and accession number(s) can be found below: European Nucleotide Archive (ENA) repository, accession number PRJEB55703.

## Ethics statement

Ethical review and approval was not required for the animal study because the animals used in this work were not subjected to any experimental protocol and were a part of the routine procedures of a commercial hatchery facility. All animals were handled by the fish farm employees, the euthanasia method used was an anaesthetic overdose of the commercial anaesthetic Aquacen benzocaine 200 mg/ml (CENAVISA, S.L., Spain) following SEA EIGHT’s Veterinary Plan. According to the Portuguese legislation DL N° 113/2013, this work is exempted from the need for ethical approval. All methods are reported in accordance with ARRIVE guidelines.

## Author contributions

DA, MS, CM, IB, and AM had substantial contributions in the conception and design of the work. DA and IB were responsible for the acquisition of the samples. DA and MS for the analysis. DA, MS, CM, and AM in the interpretation of the data. DA drafted the first manuscript. MS, CM, IB, and AM revised it critically. MS, CM, and AM provided final approval for publication of the content. All authors contributed to the article and approved the submitted version.

## Funding

This work was funded by the project ATLANTIDA (NORTE-01-0145-FEDER-000040), supported by the North Portugal Regional Operational Program (NORTE2020), under the PORTUGAL 2020 Partnership Agreement and through the European Regional Development Fund (ERDF). DA was supported by the Ph.D. grant with the reference PD/BDE/135542/2018, and Safiestela Sustainable Aquafarming Investments, S.A. (part of the SEA EIGHT group). MS was supported by the project 39948_FeedMi for this work, supported by Portugal and the European Union through FEDER/ERDF, CRESC Algarve 2020 and NORTE 2020, in the framework of Portugal2020. AM and CM were supported by the Strategic Funding UIDB/04423/2020 and UIDP/04423/2020 through national funds provided by FCT and ERDF.

## Conflict of interest

DA and IB were employed by Safiestela S.A., SEA EIGHT group at the time of the study.

The remaining authors declare that the research was conducted in the absence of any commercial or financial relationships that could be construed as a potential conflict of interest.

## Publisher’s note

All claims expressed in this article are solely those of the authors and do not necessarily represent those of their affiliated organizations, or those of the publisher, the editors and the reviewers. Any product that may be evaluated in this article, or claim that may be made by its manufacturer, is not guaranteed or endorsed by the publisher.
