# Supplementary material for: Sole microbiome progression in a hatchery life cycle, from egg to juvenile
Source: Front Microbiol. 2023 Jun 26;14:1188876. doi: 10.3389/fmicb.2023.1188876 (PMC10331008; doi:10.3389/fmicb.2023.1188876)
Supplement: Supplementary file 1 [file Data_Sheet_1.DOCX]

Supplementary Material

Sole microbiome progression in a hatchery life cycle, from egg to juvenile

Diana Bastos Almeida^1,2,3^, Miguel Semedo^2*^, Catarina Magalhães^2,4^, Isidro Blanquet^3^, Ana Paula Mucha^2,4^

^1^ ICBAS – Instituto de Ciências Biomédicas Abel Salazar, University of Porto, Porto, Portugal

^2^ CIIMAR - Interdisciplinary Centre of Marine and Environmental Research, University of Porto, Matosinhos, Portugal

^3^ SEA EIGHT - Safiestela S.A, Estela, Portugal.

^4^ FCUP – Faculty of Sciences, University of Porto, Porto, Portugal.

*** Correspondence:** Miguel Semedo: msemedo@ciimar.up.pt

# Supplementary Figures

**Supplementary Figure 1.** Schematic diagram of the recirculating aquaculture system present in the weaning (WE) and pre-ongrowing systems (PO).

**Supplementary Figure 2.** Relative phylum composition of the prokaryotic community ordered by age and sample type.

**Supplementary Figure 3.** Relative genus composition of the prokaryotic community (abundance >0.01) ordered by age and sample type.

**Supplementary Figure 4.** Species richness (Observed ASVs) and alfa-diversity (Shannon index), grouped by age (A) and by sample type (B).
