# Supplementary material for: Sole microbiome progression in a hatchery life cycle, from egg to juvenile
Source: Front Microbiol. 2023 Jun 26;14:1188876. doi: 10.3389/fmicb.2023.1188876 (PMC10331008; doi:10.3389/fmicb.2023.1188876)
Supplement: Supplementary file 2 [file Table_1.DOCX]

Supplementary Material

The progression of the sole microbiome in a hatchery life cycle, from egg to juvenile

Diana Bastos Almeida^1,2,3^, Miguel Semedo^2*^, Catarina Magalhães^2,4^, Isidro Blanquet^3^, Ana Paula Mucha^2,4^

^1^ ICBAS – Instituto de Ciências Biomédicas Abel Salazar, University of Porto, Porto, Portugal

^2^ CIIMAR - Interdisciplinary Centre of Marine and Environmental Research, University of Porto, Matosinhos, Portugal

^3^ SEA EIGHT - Safiestela S.A, Estela, Portugal.

^4^ FCUP – Faculty of Sciences, University of Porto, Porto, Portugal.

*** Correspondence:** Miguel Semedo: msemedo@ciimar.up.pt

**Table S1:** Temperature (ºC), pH, and salinity (‰) recorded in the hatchery at collection dates (DAH - days after hatching).

| **DAH** | **-2** | **2** | **14** | **49** | **119** | **146** |
| --- | --- | --- | --- | --- | --- | --- |
| **System** | Egg | Larvae | Larvae | Weaning | Pre-Ongrowing | Pre-Ongrowing |
| **Temperature** | 19.7 | 19.1 | 19.5 | 19.7 | 20.5 | 19.9 |
| **pH** | 8.48 | 8.41 | 8.43 | 7.75 | 7.68 | 7.75 |
| **Salinity** | 35 | 35 | 36 | 35 | 36 | 35 |
