# Supplementary material for: Sole microbiome progression in a hatchery life cycle, from egg to juvenile
Source: Front Microbiol. 2023 Jun 26;14:1188876. doi: 10.3389/fmicb.2023.1188876 (PMC10331008; doi:10.3389/fmicb.2023.1188876)
Supplement: Supplementary file 5 [file Table_4.DOCX]

Supplementary Material

The progression of the sole microbiome in a hatchery life cycle, from egg to juvenile

Diana Bastos Almeida^1,2,3^, Miguel Semedo^2*^, Catarina Magalhães^2,4^, Isidro Blanquet^3^, Ana Paula Mucha^2,4^

^1^ ICBAS – Instituto de Ciências Biomédicas Abel Salazar, University of Porto, Porto, Portugal

^2^ CIIMAR - Interdisciplinary Centre of Marine and Environmental Research, University of Porto, Matosinhos, Portugal

^3^ SEA EIGHT - Safiestela S.A, Estela, Portugal.

^4^ FCUP – Faculty of Sciences, University of Porto, Porto, Portugal.

*** Correspondence:** Miguel Semedo: msemedo@ciimar.up.pt

**Table S4:** Results for the Adonis test for beta group significance with a Bray-Curtis distance matrix. The null hypothesis is that there is no interaction between our test variables and prokaryotic diversity. The null hypothesis that groups have the same dispersion was also tested with the homogeneity of dispersion.

| **Variable** | **p-value** | **R^2^** | **Homogeneity of dispersion test** |
| --- | --- | --- | --- |
| Age | <0.001 | 0.112 | 0.019 |
| Sample_type | 0.030 | 0.225 | 0.041 |
| Stage | 0.024 | 0.111 | 0.097 |
| System | <0.001 | 0.248 | 0.001 |
